# Editorial: Outcome prediction in pituitary adenomas

**DOI:** 10.3389/fendo.2022.990374

**Published:** 2022-08-23

**Authors:** Nazanin Hosseinkhan, Mohammad E. Khamseh, Margherita Bignami, Alessandro Giuliani

**Affiliations:** ^1^ Endocrine Research Center, Institute of Endocrinology and Metabolism, Iran University of Medical Sciences, Tehran, Iran; ^2^ Environment and Health Department, Istituto Superiore di Sanità, (Italian NIH), Rome, Italy

**Keywords:** pituitary adenoma, outcome prediction, molecular biomarkers, machine learning, deep learning

Pituitary adenomas (PAs) are typically slowly progressing tumors of the anterior pituitary gland. They represent a wide spectrum of clinical behavior including invasion, regrowth, and persistence of hormone hypersecretion, causing significant morbidity and mortality. Clinical heterogeneity, both between and within each subtype, is reported among various pituitary adenomas, posing a unique predictive challenge.

Prediction of tumor behavior following surgical resection is currently made by using clinicopathological factors. In addition, immunohistochemical(IHC) evaluation of a variety of markers such as Ki-67, p21, p27, p53, and EGFR (https://link.springer.com/article/10.1007/s12022-018-9563-2,https://www.ncbi.nlm.nih.gov/pmc/articles/PMC6867968/, https://aacrjournals.org/cancerres/article/80/17/3451/645801/p27-as-a-Transcriptional-Regulator-New-Roles-in) are commonly used to predict long term outcomes of PAs. In a recent systematic review on molecular alterations in invasive non-functional pituitary adenoma (NFPA) (https://pubmed.ncbi.nlm.nih.gov/35711030/), several deregulated genes, proteins, and miRNAs involved in invasiveness/metastasis-associated pathways were detected as possible prognostic biomarkers. Interestingly, these ‘signatures’ are shared by the majority of malignant cancers and include epithelial-mesenchymal transition (EMT), angiogenesis, and hypoxia processes, which in turn hold promises to be used in practice to distinguish invasive from non-invasive NFPA. These include CDH1, CCNB1, PTTG1, SNAI1, and SNAI2 involved in EMT, VEGFA, FLT1, PTTG1, CTNNB1, CCND1, and MYC contributing to angiogenesis, and FLT1, SLC2A1, and VEGFA associated with hypoxia. It is worth noting that none of the identified deregulated genes, proteins, or miRNAs was frequently observed in NFPA patients. This finding suggests a great heterogeneity among patients suffering from invasive NFPA, while simultaneously implying that deregulated signaling pathways and processes (and more generally the alteration of the gene expression correlation structure) play a more critical role than the quantitative change of a few specific biomarkers. In other words, instead of considering a single biomarker, for example, the high expression of proliferative marker Ki-67, a panel of biomarkers contributing to different pathways (together with their relative balance) would be a more reliable strategy for outcome prediction in invasive NFPA.

Another valuable data source that remarkably helps for outcome prediction in PA is the conventional Magnetic Resonance Imaging (MRI) data, which is the gold standard method for localization and exploring features associated with invasiveness such as extrasellar extension and cavernous sinus invasion. These characteristics help clinicians to predict progression/recurrence (P/R) after tumor resection (https://www.frontiersin.org/articles/10.3389/fonc.2022.813806/full).

The availability of large genomics, transcriptomics, and proteomics data sets besides imaging features in PA, has recently provided the opportunity to apply machine learning (ML) algorithms to improve outcome prediction. Different types of supervised learning classes of ML algorithms are commonly used to build predictive models. These algorithms, using minimizing the discrepancy between the known outcome and the one predicted by the automatic analysis of a feature vector, generate an optimal weighted set of predictive features present in the training data set which can then be generalized to unseen data (test set) (https://link.springer.com/referenceworkentry/10.1007/978-1-4419-1428-6_451). Different supervised learning algorithms can be used for outcome prediction based on the outcome types; i.e., binary or multi-class outcomes. Examples of binary outcomes would be the prediction of the response to a given treatment program or surviving to a specific time point from diagnosis (e.g., 5 years). Multi-class outcomes include situations where different types (more than two) of outcomes are possible (https://pubmed.ncbi.nlm.nih.gov/34927670/).

A large number of ML/Deep learning (DL) algorithms have been already employed to predict different outcomes in PA including prediction of response to medical treatment as well as transsphenoidal surgery (TSS) (https://www.ncbi.nlm.nih.gov/pmc/articles/PMC8733587/). Here we will focus on four articles appearing in this Research Topic, as examples of employing ML/DL algorithms to predict outcomes in PA.

Radiomics is a machine learning method that is applied for the extraction of a large number of features from medical images that cannot be recognized by traditional image inspection methods. The recently published study in the “*Frontiers in Oncology*” journal is an example of these types of studies in which Zhang et al. employed a radiomic approach on preoperative and postoperative MRI follow-ups of 50 NFPA patients. SVM (Support Vector Machine, a classical machine intelligence method based on distance metrics) algorithm was selected as the most efficient in the P/R after surgery prediction. The radiomics model could differentiate P/R from non-P/R NFPAs with an accuracy of 82% and the Area Under Curve (AUC) of 0.78 (https://www.ncbi.nlm.nih.gov/pmc/articles/PMC7775655/). Still more relevant, the method gave rise to a non-redundant set of ‘most predictive features’ allowing for a clinical explanation of the observed results.

In another radiomics study, Staartjes et al. used a DL neural network algorithm on radiological and procedural variables of 140 patients with PA who underwent endoscopic transsphenoidal surgery to predict Gross-Total Resection (GTR) (https://pubmed.ncbi.nlm.nih.gov/30453454/). They highlighted tumor diameter along three orthogonal axes, tumor volume, and the R ratio between maximum adenoma diameter and ICD C4 horizontal segment, as the most predictive features. The results showed an improvement in the prediction of tumor behavior compared to the standard Knosp classification using a conventional statistical approach to predicting binary outcomes.

In another published article in the “*Frontiers in Endocrinology*” journal, under the topic of “*pituitary adenoma: targeted therapy*”, Huber et al, used a combination of different supervised classifiers, a gradient boosting machine known as the *super learner*. The model’s outcome was evaluated by two performance metrics: the area Under the Receiver Operating Curve (AUROC) and the Matthews correlation coefficient (MCC). Their model was trained to predict the dependence risk on long-term administration of dopamine agonists (DAs) in 86 prolactinoma patients subjected to upfront surgery and to control hyperprolactinemia at early and long-term follow-up. Their results showed that baseline prolactin levels are the most important outcome predictor at early follow-up.

In another study conducted on 400 PA individuals, four different ML models including naïve Bayes, logistic regression with elastic net (LR-EN) regularization, support vector machine with linear kernel, and random forest, were evaluated for predicting early outcomes. The results showed that sodium dysregulation, age, obesity, Cushing’s disease, and sex are the most predictive features for stratifying patients into: with/without poor postoperative outcomes (https://thejns.org/focus/view/journals/neurosurg-focus/45/5/article-pE8.xml).

It must be noted that adequate sample size is a prerequisite for conducting ML/DL-based studies because it helps to select a sufficient number of features to avoid overfitting which results in the more generalizability of the predictive model (https://www.ncbi.nlm.nih.gov/pmc/articles/PMC6611436/). Another source of overfitting is when the MRI images were collected from a single laboratory or prepared with the same protocol. Therefore, collecting samples from diverse sources using different techniques will help to avoid overfitting in ML/DL algorithms. Genomic features are commonly used to assess the reliability of ML/DL predictive models.

Overall, the articles included in this Research Topic showed that multidisciplinary approaches are very promising for outcome prediction in PA so opening a new avenue of research in the field fostered by the close interaction among computational and biomedical scientists.

## Author contributions

MK conceptualized the topic of the manuscript. NH wrote the original draft of the manuscript. MK, AG, and MB reviewed and edited the manuscript. All authors contributed to the article and approved the submitted version.

## Conflict of interest

The authors declare that the research was conducted in the absence of any commercial or financial relationships that could be construed as a potential conflict of interest.

## Publisher’s note

All claims expressed in this article are solely those of the authors and do not necessarily represent those of their affiliated organizations, or those of the publisher, the editors and the reviewers. Any product that may be evaluated in this article, or claim that may be made by its manufacturer, is not guaranteed or endorsed by the publisher.

